# Effects of Dietary Bisphenol A on the Reproductive Function of Gilthead Sea Bream (*Sparus aurata*) Testes

**DOI:** 10.3390/ijms20205003

**Published:** 2019-10-10

**Authors:** Isabel Forner-Piquer, Ioannis Fakriadis, Constantinos C Mylonas, Fabiana Piscitelli, Vincenzo Di Marzo, Francesca Maradonna, Josep Calduch-Giner, Jaume Pérez-Sánchez, Oliana Carnevali

**Affiliations:** 1 Dipartimento Scienze della Vita e dell’Ambiente, Università Politecnica delle Marche, Via Brecce Bianche, 60131 Ancona, Italy; isabel.forner.piquer@gmail.com (I.F.-P.); f.maradonna@staff.univpm.it (F.M.); 2 Institute of Marine Biology, Biotechnology and Aquaculture, Hellenic Center for Marine Research, P.O. Box 2214, Heraklion, 71003 Crete, Greece; fakriadis@hcmr.gr (I.F.); mylonas@hcmr.gr (C.C.M.); 3 Endocannabinoid Research Group, Istituto di Chimica Biomolecolare, Consiglio Nazionale delle Ricerche, Via Campi Flegrei, 80078 Pozzuoli, Italy; fpiscitelli@icb.cnr.it (F.P.); vdimarzo@icb.cnr.it (V.D.M.); 4 Canada Excellence Research Chair on the Microbiome-Endocannabinoidome Axis in Metabolic Health, Université Laval, Quebec City, QC G1V 0A6, Canada; 5 Nutrigenomics and Fish Endocrinology Group, Institute of Aquaculture Torre de la Sal (IATS-CSIC), 12595 Ribera de Cabanes, Castellón, Spain; j.calduch@csic.es (J.C.-G.); jaime.perez.sanchez@csic.es (J.P.-S.)

**Keywords:** BPA, endocannabinoids, gonads, gilthead sea bream

## Abstract

Bisphenol A (BPA), a known endocrine disrupting chemical (EDC), was administered by diet to gilthead sea bream (*Sparus aurata*) in order to study its effects on the endocannabinoid system (ECS) and gonadal steroidogenesis. 2-year-old male gilthead sea bream were fed with two different concentrations of BPA (LOW at 4 and HIGH at 4000 µg/kg body weight for 21 days during the reproductive season. Exposure to 4000 µg BPA/kg bw/day (BPA HIGH) reduced sperm motility and altered the straight-line velocity (VSL) and linearity (LIN). Effects on steroidogenesis were evident, with testosterone (T) being up-regulated by both treatments and 11-ketotestosterone (11-KT) down-regulated by BPA HIGH. Plasma levels of 17β-estradiol (E_2_) were not affected. The Gonadosomatic Index (GSI) increased in the BPA HIGH group. Interestingly, the levels of endocannabinoids and endocannabinoid-like compounds were significantly reduced after both treatments. Unpredictably, a few changes were noticed in the expression of genes coding for ECS enzymes, while the receptors were up-regulated depending on the BPA dose. Reproductive markers in testis (leptin receptor (*lepr*), estrogen receptors (*era*, *erb*), progesterone receptors (*pr*) and the gonadotropin releasing hormone receptor (*gnrhr*)) were up-regulated. BPA induced the up-regulation of the hepatic genes involved in oogenesis (vitellogenin (*vtg*) and zona pellucida 1 (*zp1*)).

## 1. Introduction

Since 1950, Bisphenol A (BPA, 2,2-bis-(4-hydroxyphenyl)-propane; CAS No. 80-05-7) has been one of the key materials used for the manufacture of polycarbonate plastics and epoxy resins [1]. In a recently published report, the global BPA market is expected to reach around 7348 thousand tons by the end of 2023 [2], making this compound one of the top manufactured and used chemicals. Consequently, BPA has become ubiquitous, being identified in multiple environmental matrices [3,4]. Indeed, BPA has the capacity to migrate from products such as metallic food cans, polycarbonate baby bottles or reusable water bottles, to water or canned food [5]. Although laboratory-based studies have shown evidence of the low ability of BPA to concentrate from the water to the biota and a rapid elimination of this component in fish occurs [3,6,7], it has been detected in tissues of different wildlife species, suggesting that wild organisms are chronically exposed to this contaminant [3,8,9]. 

The endocrine disrupting activities induced by BPA in teleosts can be summarized in: (1) alterations in the estrogen / androgen ratio [10,11]; (2) alterations in gonadal development and gamete quality [1,10,11,12,13,14]; (3) induction of hepatic vitellogenin production [1,15,16] and (4) epigenetic effects [17,18]. Even though BPA has been reported to exhibit lower estrogenic activity than 17β-estradiol [19], its disrupting potency has also been demonstrated to occur at environmental concentrations in fish [1,11,12,17,20].

On the other hand, the implication of the Endocannabinoid System (ECS) in both mammalian and non-mammalian species reproductive events is also well documented, being a lipid signaling system pivotal for the success of reproductive performance and closely related to the levels of sex-steroids [21]. The ECS is present in the gonads of teleosts, among other tissues, as it has been previously reported in zebrafish *Danio rerio* [22], goldfish *Carassius auratus* [23] and gilthead sea bream *Sparus aurata* [24], with a functional relevance in teleost reproduction [23,25]. Local mediators, namely the endocannabinoids (Anandamide (AEA) and 2-arachidonoylglycerol (2-AG)), their membrane receptors (endocannabinoid receptor type I (CNR1) and type II (CNR2), vanilloid receptor (TRPV1)) and the enzymatic machinery that regulates the levels of the endocannabinoids, compose the ECS, as thoroughly described in several review articles [26,27,28]. Thus, the main goal of the present study was to elucidate whether BPA consumed with food might alter the gonadal ECS and steroidogenesis and, in turn, sperm characteristics during the first reproductive cycle of male gilthead sea bream.

## 2. Results

### 2.1. BPA Increased GSI and Altered Sperm Quality 

**Figure 1:**
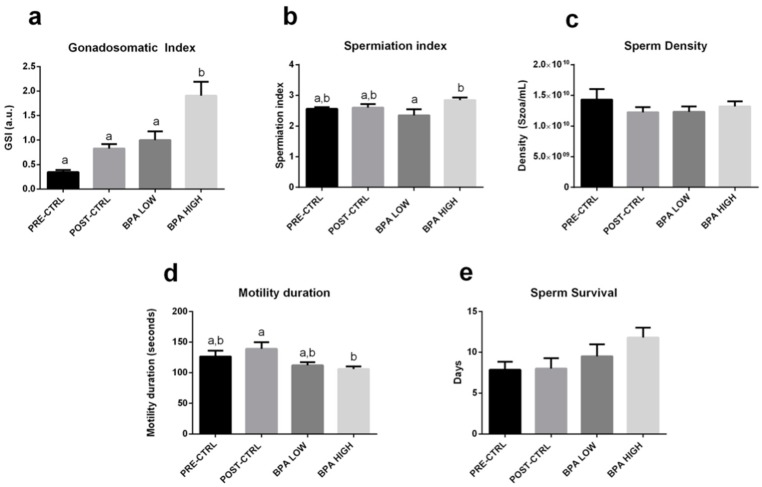
Mean ± SEM of gilthead sea bream (**a**) Gonadosomatic Index (GSI)**;** (**b**) Spermiation index; (**c**) Sperm density (szoa/mL); (**d**) Sperm motility duration (sec); (**e**) Sperm survival (days). Letter superscripts above the means indicate significant differences among treatments (one-way ANOVA, Tukey’s post hoc test, *p* < 0.05). Absence of letters means no significant differences among groups. Spermiation index was evaluated as S0 = no milt released, S1 = only a drop of milt released after multiple stripping attempts, S2 = milt easily released after the first stripping attempt, S3 = copious amounts of milt flowing with the slightest pressure.

**Figure 2:**
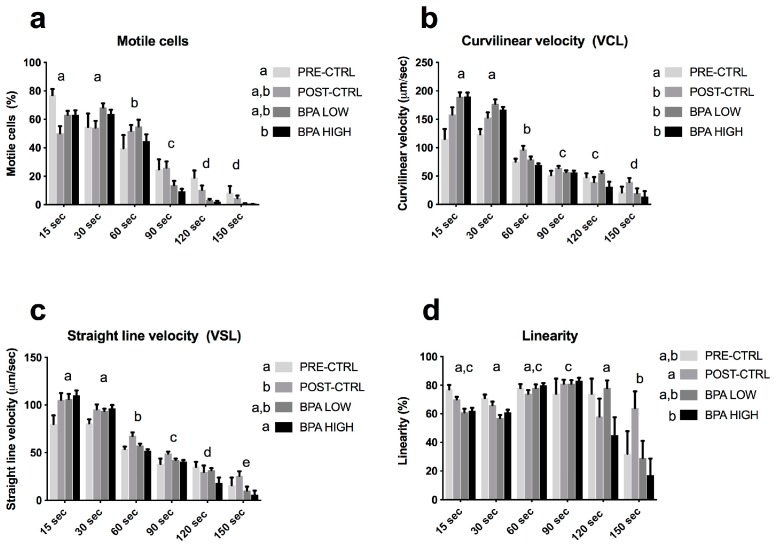
Computer-Assisted Sperm Analysis (CASA) of gilthead sea bream milt. Mean ± SEM of (**a**) percentage of motile cells; (**b**) VCL as µm/sec; (**c**) VAP as µm/sec; (**d**) VSL as µm/sec and (**e**) linearity in percentage (%). Statistically significant differences (two-way ANOVA, Tukey’s post hoc test, *p* < 0.05) are indicated by different letter superscripts among treatments (in the legend, next to the experimental groups) and sample times (above the columns).

Only the highest concentration of BPA increased the GSI (Figure 1a). No effects were found on the spermiation index (Figure 1b), sperm density (Figure 1c) and survival (Figure 1e). However, sperm motility duration was significantly decreased in the BPA HIGH group with respect to the POST-CTRL group (Figure 1d). Whilst the percentage of motile cells (Figure 2a) and the curvilinear velocity (Figure 2b) were not affected by the treatments with respect to the POST-CTRL group, the straight-line velocity (Figure 2c) and the linearity (Figure 2d) were significantly altered by the BPA HIGH treatment regarding the POST-CTRL animals.

### 2.2. Gonadal Morphology 

**Figure 3:**
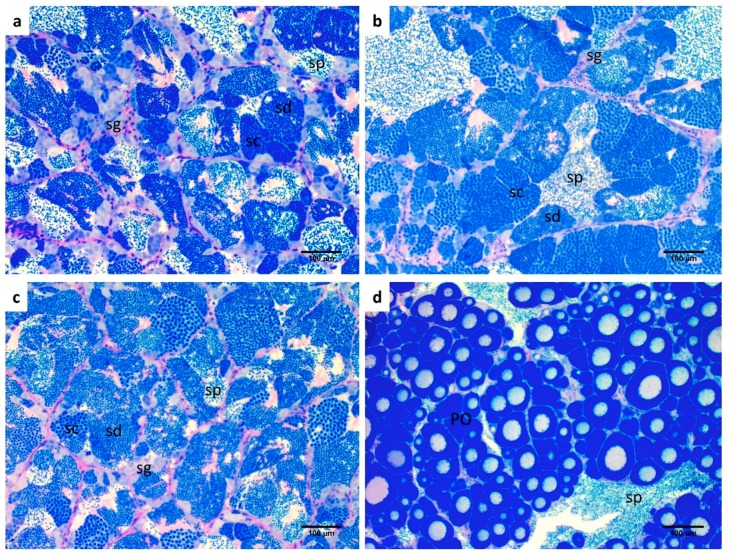
Histological images of gilthead sea bream gonads. Testicular tissue from (**a**) POST-CTRL (**b**) BPA LOW; (**c**) BPA HIGH and (**d**) ovotestis containing an ovarian part with primary oocytes (PO). Testicular part with spermatozoa (sp); sd: spermatids, sg: spermatogonia, sc: spermatocytes. Scale bar: 100 µm.

The histology of the testes was performed in order to assess whether BPA induced morpho-pathological alterations in the gonads. No differences with respect to the control group (Figure 3a) were observed for each experimental groups (Figure 3b,c). All individuals were in the peak of the spawning season and all cell stages, from spermatogonia to spermatozoa, were present. Regarding the small ovarian part of the testes (Figure 3d), all groups exhibited previtellogenic oocytes.

### 2.3. Altered Endocannabinoid and Endocannabinoid-Like Mediator Levels in the Testis 

**Figure 4:**
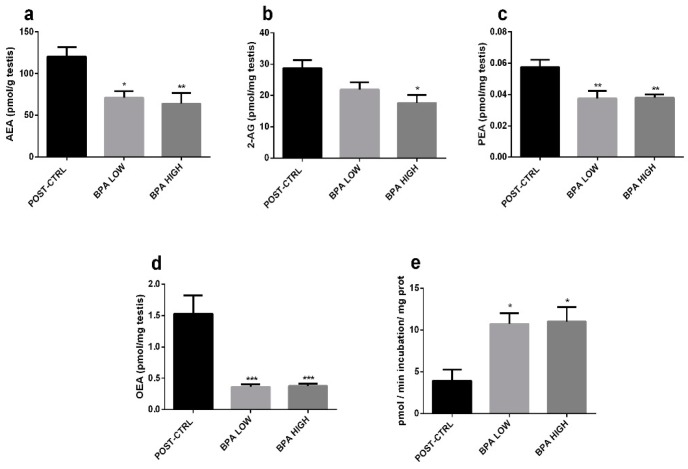
(**a**–**d**) Testicular Endocannabinoid levels. Data reported as mean ± SEM. Asterisks above each column denote significant differences between POST-CTRL and BPA groups (one-way ANOVA, Dunnett’s multiple comparisons test, * (*p* < 0.05), ** (*p* < 0.01), *** (*p* < 0.001)). AEA expressed as pmol/gr tissue, while 2-AG, PEA and OEA as pmol/mg tissue. (**e**) FAAH enzymatic activity in testes. Data reported as mean ± SEM of pmol/minutes incubation/mg protein. Asterisks above each column indicate significant differences between POST-CTRL and BPA group (one-way ANOVA, Dunnett’s multiple comparisons test, * (*p* < 0.05)).

Levels of AEA (Figure 4a), and endocannabinoid-like mediators PEA and OEA (Figure 4c,d) were significantly reduced in both treated groups, while the other endocannabinoid, 2-AG, was decreased only in the BPA HIGH individuals (Figure 4b). In agreement with the observed levels of AEA, PEA and OEA, the activity of the enzyme in charge of the hydrolysis of these biochemically related mediators, i.e., fatty acid amide hydrolase (FAAH), was significantly increased in the BPA-exposed fish (Figure 4e).

### 2.4. Sex Steroid Levels 

**Figure 5:**
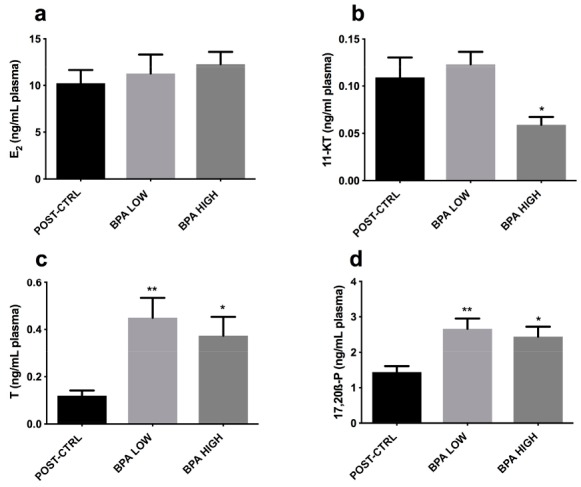
Mean ± SEM plasma levels of (**a**) Estradiol (E_2_), (**b**) 11-Ketosterone (11-KT), (**c**) Testosterone (T) and (**d**) 17,20β-P. Asterisks above each column denote significant differences between POST-CTRL and BPA groups (one-way ANOVA, Dunnett’s multiple comparisons test, * (*p* < 0.05), ** (*p* < 0.01)).

Treatment with BPA did not alter the plasma levels of E_2_ (Figure 5a), however, the 11-KT ones were significantly reduced in the BPA HIGH group (Figure 5b). Both concentrations of BPA triggered an increase in T and 17, 20β-P plasma levels (Figure 5c,d).

### 2.5. Modifications at The Transcriptomic Level in The Male Sea Bream Liver and Testis 

**Figure 6:**
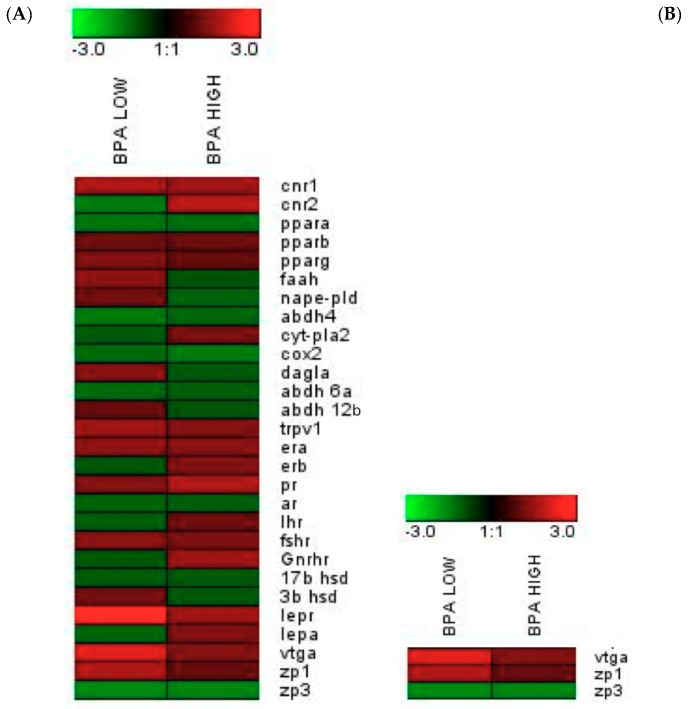
Gene expression heat maps in the testes (**A**) and the liver (**B**). Scales of colors represent fold-change values in relation to the POST-CTRL group (green, down-regulation; red, up-regulation).

qPCR-arrays were performed for genes coding for ECS components and reproductive signals in samples form gilthead sea bream liver and testis. The results obtained are reported in Table 1 for the testis and Table 2 for the liver. Fold-changes of the target genes are represented in heatmaps (Figure 6A,B). Basically, in the testis, for the BPA LOW group, genes coding for the endocannabinoid receptor type I (*cnr1*), vanilloid receptor (*trpv1*), leptin receptor (*lepr*) and estrogen receptor (*era*) were up-regulated and only the α,β-hydrolase-4 (*abdh4*), an alternative biosynthetic enzyme for AEA, PEA and OEA, was down-regulated. Regarding the effects of BPA HIGH, the mRNA levels of the endocannabinoid receptor II (*crn2*), estrogen receptor (*erb*), progesterone receptor (*pr*) and gonadotropin-releasing hormone receptor (*gnrhr*) were increased. In the liver, BPA LOW treatment induced an up-regulation of the genes coding for vitelogenin (*vtg*) and zona pellucida protein (*zp1*).

## 3. Discussion

The ubiquitous presence of BPA in a large number of items, including canned food, poses a question around the safety of this compound, which is reported to behave as an estrogen-like chemical. In the present study, BPA was administered by food to adult male gilthead sea bream. Gilthead sea bream represents an interesting model for the study of EDCs due to its sex reversion [29]. For this reason, in the present study, gilthead sea bream were sampled during their first reproductive cycle, when all individuals still function as males. However, unraveling the activity induced by BPA is generally puzzling, due to the tissue-specific disruptive activities, species-specific sensibilities, and dose-dependent effects of this compound [11,30]. Indeed, BPA can differentially affect teleost male GSI depending on the dose administered and duration of treatment, making it difficult to draw conclusions regarding its effects on GSI, which in turn, would consequently not be considered a good indicator for BPA effects [11,31]. However, increased GSI was reported with different BPA concentrations administered for 43 days to male fathead minnows *Pimephales promelas* [14], and with 5 µg BPA/L to male rare minnows *Gobiocypris rarus* [32]. Controversially, chronic treatments with environmental relevant concentrations of BPA did not alter GSI in male goldfish [11,12], in brown trout *Salmo trutta* male at 50 µg/L [15], in male medaka *Oryzias latipes* at higher concentrations [33] and in rare minnows treated with 15 and 225 µg/L [34]. Nevertheless, as some previtellogenic oocytes were observed, we hypothesized that the GSI rise may have been caused by the increasing size of the ovarian part, particularly since all groups showed the same spermiation index and similar histological results. Indeed, as previously reported [14], EDC effects on adult teleosts not only depend on the type of EDC, but also on the duration and timing of the exposure. Furthermore, we need to take into account that when BPA is orally administered and not diluted in water, the kinetics could be different due to low BPA absorption or fast metabolism [35].

BPA did not induce any meaningful change in E_2_ levels in our experiments. This finding was in agreement with the unaltered levels of this hormone found in male goldfish treated with BPA [12]. Additionally, decreased levels of E_2_ have been reported after treatment with environmental concentrations of BPA in male carps [10] and a reduction of E_2_ metabolism was described in immature lake trout *Salvelinus namaycush* [36]. Though E_2_ was unaffected, mRNA levels of *vtg* and *era* were increased in the BPA LOW group; indeed, in male goldfish, 0.2 µg BPA /L increased the expression of *erb1* mRNA in the liver but not *vtg* mRNA; however, a higher dose of BPA stimulated both *era* and *erb*, up-regulating *vtg* [11]. On the other hand, *erb* was only augmented by the highest dose of BPA tested, underlying a dose-related action of the compound on ERα and ERβ expression [37]. It was unexpected to find such levels of T, which was probably due to the modulation of aromatase activity by BPA [11,38]. In this sense, higher levels of T (although not statistically significant) have been found in male carps treated with 1 µg BPA/L [10], and increased levels of T in male rare minnows treated with 225 µg/L [38]. Instead, the levels of 11-KT, the active androgen of teleost, were reduced in the present study, similarly to male goldfish exposed to 4.5 and 11 µg BPA/L for 20 days [12].

In spite of these hormonal changes, no morphological alteration was observed in the testis, even if these steroids were supposed to decrease during spermiation [39], suggesting time-dependent levels, as previously reported in gilthead sea bream treated with 5 µg EE_2_/g food [40]. The study of [40] evidenced an increase in plasma E_2_ and T levels after seven days, followed by a decrease of T and 11-KT levels after 28 days. Furthermore, taking into account that injections of exogenous E_2_ switch functional testis to the post-spawning stage, [41] suggested that higher levels of this sex hormone were needed to orchestrate testicular regression.

In the case of 17,20β-P, previous studies evidenced that this hormone stimulated milt production and sperm motility in male trout [42] and triggers oocyte maturation in female ovaries. However, no morphological difference was observed in the BPA groups. In spite of the clearly changed levels of sex steroids, it seems that that alteration and/or the exposure time was likely to be insufficient to induce morphological changes in the testis, in agreement with studies showing pathological testicular alterations after longer BPA exposure times [14,43].

Comparing the steroidogenic effects of BPA with that induced by DiNP in our previous ones [44], similar results were found regarding the lower levels of 11-KT associated with higher levels of T. Unlike DiNP, BPA did not modify the plasma E_2_ levels. However, while DiNP reduced the levels of 17,20β-P, BPA increased them. The differences found between these two contaminants showed that both contaminants were able to alter steroidogenesis but in different ways, suggesting a different way of controlling steroidogenesis.

Focusing on sperm, previous publications have indicated that different dosages of BPA decrease sperm velocity in teleosts [12,13,34,45], as in the BPA HIGH group. On the other hand, different trends have been observed for sperm concentration: while in the brown trout it was reduced [13]; [34] reported unaltered sperm concentrations for rare minnows exposed to 15 and 225 µg BPA/L, which was in agreement with our results. As it was previously reported [44], differences in sperm parameters were found between PRE-CTRL and POST-CTRL groups, likely associated with the different sampling time. PRE-CTRL group was sampled during January, and POST-CTRL in late February, during the peak of the spawning season. This could explain the significant differences found in sperm quality between these two groups [46].

The importance of the ECS in male reproduction was first documented in sea urchins [47]. Thereafter, several studies evidenced the role of this system in both male and females of several species reproduction [25,48,49]. Indeed, the ECS interferes with the brain-pituitary-gonadal axis, affecting Sertoli and Leydig cells, germ cell differentiation, sperm motility and the acrosome reaction [27]. Additionally, a cross-talk among the ECS and sex steroids exists [21,50]. In mouse Sertoli cells, E_2_ via ER binds the ERE (estrogen response element) sites of the FAAH promoter, thereby stimulating *faah* expression and FAAH activity, and subsequently decreasing AEA levels and protecting the testis from AEA-induced apoptosis [50,51]. In the present work, treatment with BPA did not increase E_2_ levels or *faah* expression, but enhanced FAAH enzymatic activity. Consequently, reduced levels of AEA and AEA-related compounds (PEA and OEA) were found. The levels of 2-AG were also reduced, in agreement with previous data indicating that this endocannabinoid may act as an alternative FAAH substrate [52]. In comparison to the results of Grimaldi and Rossi mentioned above, who reported increased FAAH activity concomitantly with *faah* mRNA expression in Sertoli cells after E_2_ treatment; BPA seemed to execute an estrogen-like effect on FAAH activity rather than *faah* expression in gilthead sea bream. Therefore, two hypotheses were formulated: 1) BPA may exert a different effect at the transcriptional and translational/post-translational levels, or 2) transcription and translation can be uncoupled in testes [53,54]. The effects of BPA on ECS were similar to that observed with DiNP [44] suggesting a similar ECS control by these two contaminants.

Finally, a correct endocannabinoid “tone” is essential for the successful progression of spermatogenesis. In fact, lower levels of AEA and 2-AG have been found in the sperm of infertile men [55], suggesting that further fertilizing effects may be observed in gilthead sea bream treated with BPA, jointly with the changed sperm parameters mentioned before. The mRNA expression levels of some endocannabinoid targets and related mediators (*cnr1*, *cnr2* and *trpv1*) were slightly increased by one of the two doses of BPA, possibly as a compensatory response to the reduction of AEA, 2-AG, PEA and OEA. These receptors have all been implicated in various aspects of the physiopathology of sperm cells and oocytes [55,56,57,58,59]. The role of these receptors in the potentially ECS-mediated effects of BPA constitutes an interesting line of research that merits further consideration in future studies.

**Figure 7:**
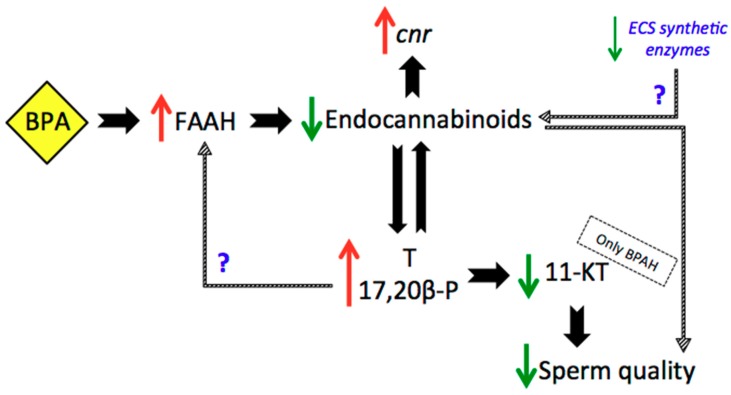
Summarized results. Abbreviations: BPAH (BPA HIGH group), FAAH (Fatty acid amide hydrolase), *cnr* (endocannabinoid receptors), T (testosterone), 11-KT (11-Ketotestosterone). Red arrows: increase, green arrows: decrease.

In summary (Figure 7), the effects of BPA—one of the most worldwide manufactured EDCs—on the reproductive physiology of different species has been largely studied. However, the effects of BPA are still difficult to accurately predict due to their variability depending on the dose used, the duration of exposure and the developmental stage of the model used. The present study has attempted to shed some more light on the alterations induced by the temporary tolerable daily intake set by the European Food Safety Authority (EFSA) in 2015 for BPA (4 µg BPA/kg bw/day). Even if lack of a clear effect was observed concerning histological changes, spermiation index and sperm quality, significant changes were observed at the hormonal and endocannabinoid levels.

## 4. Materials and Methods

### 4.1. Fish Maintenance, Food Preparation and Animal Treatment

Two-year-old gilthead sea bream (461.09 ± 16.93 gr; 301.7 ± 10.7 mm) were maintained at the AQUALABS facilities of the Institute of Marine Biology, Biotechnology and Aquaculture of the Hellenic Centre for Marine Research (HCMR), Iraklion, Crete, Greece, following the conditions reported previously [44,60]. Gilthead sea bream are protandrous hermaphrodites, and at two years of age all individuals function as males with developed testis and previtellogenic ovaries.

After 3 months of acclimation and before the beginning of the experimentation, and following previous publications [44,60], the sperm quality parameters were assessed in January to obtain the pre-treatment data (PRE-CTRL), as explained below. Then, the fish were divided into three groups of 10 specimens (in duplicates) and left to acclimatize in the experimental tanks (2 m^3^). After the acclimation, the fish were fed with due consideration for the total amount of pellet, which was administered equal to 0.7% of the body weight (bw). The treatment was conducted for 21 days following the Organization for Economic Co-operation and Development (OECD) guidelines # 60, 78, 109 [31,61,62] via food intake, being the BPA (Sigma-Aldrich, Milano, Italy) introduced into commercial pellets (IRIDA S.A. Greece) as explained [35,44,60]. The doses administered were adjusted to give the nominal doses (4 and 4000 µg BPA/kg bw/day) according to the weight of fish shared in several meals along the day to ensure the fish ingested the proportional BPA amount.

The tanks (in duplicate) were organized as: Control group (POST-CTRL) fed with the prepared food with the vehicle (ethanol, EtOH); BPA LOW food enriched with 4 µg BPA/kg bw/day); BPA HIGH with 4000 µg BPA/kg bw/day. The lowest concentration of BPA was based on the Tolerable Daily Intake (TDI) for humans ruled by the European Food Safety Authority (EFSA), which was set from January 2015 to 4 µg of BPA/kg bw/day. 4000 µg BPA/kg bw/day was chosen as an effective concentration (EC).

The PRE-CTRL and POST-CTRL individuals were the same of those previously reported [44] since both experiments were running in parallel at the same time.

The experimentation was performed under the same conditions (tanks, temperature and photoperiod) already described elsewhere [44,60]. The experimental protocol was approved by the Greek National Veterinary Agency with the Protocol Number #255361 (29 November 2017) of the experimental facility EL91-BIOexp-04.

After 21 days (February), five fish were randomly chosen per tank and were anesthetized by clove oil (30 mg/L) following the indications of [63] and sampled. Blood was collected from the ventral vasculature behind the anal fin and stored at –20 °C for ELISA assay. Sperm was collected by gentle abdominal massage and stored at 4 °C until its evaluation. Gonads were weighed for Gonadosomatic Index (GSI) calculation ((Gonads weight (g)/Fish weight (g)) × 100). Pieces of the testes were collected and stored at –80 °C for endocannabinoid measurements and FAAH enzymatic assay. Since gilthead sea bream is a hermaphrodite species, pieces of ovo-testis were embedded in a solution of Formaldehyde:glutaraldehyde (4:1) for histological studies. For gene expression analysis, small pieces of liver and testes were stored within RNAlater (Ambion Inc., Austin, TX, USA) at 4 °C until processing.

### 4.2. Sperm Quality Evaluation

The evaluation of sperm quality was performed in the PRE-CTRL, POST-CTRL and BPA groups as previously described [44,64]. The parameters studied were the sperm density (szoa·mL^−1^), sperm survival (days), sperm motility duration (s), percentage of motile cells, Curvilinear Velocity (VCL, μm·s^−1^), Straight Line Velocity (VSL, μm·s^−1^) and Linearity (LIN, %). The analyzed spermatozoa (63–228) were within the reported range. All sperm parameters were evaluated before (PRE-CTRL) and after treatments (POST-CTRL and BPA groups).

### 4.3. Testes Histology

Histological procedures were performed as previously described [33] in the POST-CTRL and BPA groups. Briefly, pieces of ovo-testes were embedded in methacrylate resin (Technovit 7100^®^, Heraeus Kulzer, Germany). Then, they were sectioned with a microtome (Leica RM2245) and stained with Methylene Blue (Sigma-Aldrich, Munich, Germany)/Azure II (Sigma-Aldrich)/Basic fuchsine (Polysciences, Warrington, PA, USA) [65]. Being a protandrous hermaphrodite species, gilthead sea bream testes always contain a small quiescent ovarian section, which was also examined.

### 4.4. Enzyme-Linked ImmunoSorbent Assay (ELISA)

Plasma (200 µL) from POST-CTRL and BPA groups were extracted with diethyl ether following [44] procedures for quantification of testosterone (Τ), 11-Ketotestosterone (11-ΚΤ), 17β-estradiol (E_2_) and 17,20β -dihydroxy-4prengnen-3-one (17,20β-P or maturation inducing steroid, MIS), by readily established protocols [66,67].

### 4.5. RNA Extraction, cDNA Synthesis and Real-Time PCR

RNA extraction and cDNA synthesis from the liver and testis from POST-CTRL and BPA groups were conducted as previously described [60] with RNAzol solution (Sigma-Aldrich, Italy). For cDNA synthesis, the High-Capacity cDNA Archive Kit (Applied Biosystems, Foster City, CA, USA) was used with 500-ng total RNA in a final volume of 100 µL.

Real-time quantitative PCR was carried out as previously described [60] with an Eppendorf Mastercycler Ep Realplex real-time PCR system (Eppendorf, Wesseling-Berzdorf, Germany), using two 96-well PCR-array layouts designed for the simultaneous profiling of a panel of 25 genes for gonad samples and 3 genes for liver samples, selected as: i) endocannabinoid system markers: *cb1*, *cb2*, *pparα*, *pparβ*, *pparγ*, *faah*, *nape-pld*, *abdh4*, *cyt-pla2*, *cox2*, *dalgα*, *abdh6a*, *abdh12a*, *trpv1* ii) reproductive markers: *era*, *erb*, *pr*, *ar*, *lhr*, *fshr*, *gnrhr*, *17β-hsd*, *3β-hsd*, *lepr*, *lep*, *vtg*, *zp1*, *zp3*. PCR-array primers (Supplementary Table S1) were designed to obtain amplicons of 50–150 bp in length. A housekeeping gene (β-actin) and controls for general PCR performance were included on each array. The genes selected as well as the PCR results from POST-CTRL were the same from the previous published study from our group [44] to possibly compare the effects of the di-isononyl phthalate (DiNP) and BPA.

### 4.6. Concentration of Endogenous Cannabinoids (Anandamide (AEA), 2-Arachidonoylglycerol (2-AG)), Endocannabinoid-Like Mediators (Palmitoylethanolamide (PEA), Oleoylethanolamide (OEA)) and Fatty Acid Amide Hydrolase (FAAH) Enzymatic Activity in the Testis

The testes were processed and analyzed for levels of endocannabinoids and FAAH activity as previously described [60].

### 4.7. Statistical Analysis

Statistical analysis of gene expression, FAAH activity, endocannabinoid levels, ELISA and GSI were performed using one-way analysis of variance (ANOVA) followed by a Tukey’s or Dunnett’s multiple comparison test, while the CASA data was analyzed by two-way ANOVA. Results are reported as mean ± SEM (Standard Error of the Mean). The data fulfilled the condition for applying a parametric test; given the log-normalization to homogenize the variance when needed. Data in percentages were transformed to arc sinus to apply the statistical test. All statistical procedures were run using GraphPad Prism 6. Superscript letters specified statistical differences among treatments (*p* < 0.05). A superscript asterisk (*) indicated statistical significance with respect to the POST-CTRL group (* *p* < 0.05; ** *p* < 0.01; *** *p* < 0.001; **** *p* < 0.0001).

## Figures and Tables

**Table 1:** Transcriptional effects of BPA on gilthead sea bream testis. Data are reported as mean ± SEM. All data are normalized to the expression level of *cyt-pla2* POST-CTRL fish with an arbitrarily assigned value of 1. Asterisk superscript (*) indicates significant differences between the POST-CTRL group and the treatment (one-way ANOVA, Dunnett’s multiple comparisons test, * (*p* < 0.05), ** (*p* < 0.01), **** (*p* < 0.0001)). Abbreviations: *cnr1*: endocannabinoid receptor type i, *cnr2*: endocannabinoid receptor type ii, *trpv1*: transient receptor potential cation channel subfamily v member 1, *ppar*: peroxisome proliferator-activated receptor, *nape-pld*: n-acyl phosphatidylethanolamine phospholipase d, *faah*: fatty acid amide hydrolase, *daglα*: diacylglycerol lipase alpha, *cox-2*: cyclooxigenase-2, *abdh*: α/β-hydrolase, *cyt-pla2*: cytosolic phospholipase a2, *lepr*: leptin receptor, *lepa*: leptin a, *er*: estrogen receptor, *pr*: progesterone receptor, *ar*: androgen receptor, *lhr*: luteinizing hormone receptor, *fshr*: follicle-stimulating hormone receptor, *gnrhr*: gonadotropin-releasing hormone receptor, *17β-hsd*: 17β-hydroxysteroid dehydrogenase, *3β-hsd*: 3β-hydroxysteroid dehydrogenase.

Gene	POST-CTRL	BPA LOW	BPA HIGH
*cnr1*	0.38 ± 0.07	0.64 ± 0.10 *	0.60 ± 0.06
*cnr2*	0.35 ± 0.04	0.24 ± 0.04	0.63 ± 0.08 **
*ppar α*	3.33 ± 0.34	2.43 ± 0.22	2.49 ± 0.24
*ppar β*	6.41 ± 0.63	6.82 ± 0.80	6.86 ± 0.72
*ppar γ*	12.68 ± 1.69	16.14 ± 2.13	12.63 ± 1.52
*trpv1*	0.24 ± 0.03	0.37 ± 0.03 *	0.30 ± 0.03
*faah*	4.87 ± 0.49	6.81 ± 0.67	4.81 ± 0.62
*nape-pld*	22.71 ± 1.55	25.50 ± 2.50	19.29 ± 1.94
*abdh4*	3.69 ± 0.35	2.52 ± 0.35*	3.00 ± 0.19
*cyt-pla2*	1.00 ± 0.12	0.94 ± 0.12	1.22 ± 0.15
*cox2*	1.43 ± 0.18	1.13 ± 0.12	0.98 ± 0.12
*daglα*	4.39 ± 0.46	5.96 ± 0.64	4,17 ± 0.49
*abdh 6a*	4.42 ± 0.34	3.56 ± 0.35	4.00 ± 0.36
*abdh 12b*	2.56 ± 0.29	2.65 ± 0.33	2.54 ± 0.34
*lepr*	15.88 ± 2.80	51.49 ± 5.16 ****	24.75 ± 4.78
*lepa*	0.30 ± 0.03	0.25 ± 0.03	0.37 ± 0.04
*era*	3.04 ± 0.30	4.23 ± 0.34 *	4.07 ± 0.33
*erb*	11.89 ± 0.67	11.68 ± 1.13	15.33 ± 1.02 *
*pr*	4.36 ± 0.60	5.67 ± 0.66	7.57 ± 0.65 **
*ar*	8.59 ± 0.78	7.28 ± 0.89	7.72 ± 0.67
*lhr*	0.56 ± 0.06	0.50 ± 0.09	0.61 ± 0.04
*fshr*	0.46 ± 0.10	0.63 ± 0.07	0.56 ± 0.07
*gnrhr*	5.36 ± 0.81	5.11 ± 0.49	8.44 ± 0.96 *
*17b-hsd*	5.20 ± 0.50	4.75 ± 0.53	4.85 ± 0.45
*3b-hsd*	5.91 ± 0.59	6.80 ± 0.62	5.63 ± 0.76

**Table 2:** Transcriptional effects of BPA on the liver of gilthead sea bream. Data are reported as mean ± SEM. All data are normalized to the expression level of *zp1* of POST-CTRL fish with an arbitrarily assigned value of 1. Asterisk superscript (*) indicates significant differences between the POST-CTRL group and the treatment (one-way ANOVA, Dunnett’s multiple comparisons test, * (*p* < 0.05), ** (*p* < 0.01)). Abbreviations: *vtga*: vitellogenin a; *zp1*: zona pellucida—like domain—containing protein 1; *zp3*: zona pellucida sperm-binding protein 3.

Gene	POST-CTRL	BPA LOW	BPA HIGH
*vtga*	0.76 ± 0.43	1.71 ± 0.43 **	0.96 ± 0.33
*zp1*	1.00 ± 0.35	1.91 ± 0.48 *	1.00 ± 0.26
*zp3*	116.32 ± 39.82	58.51 ± 37.54	73.11 ± 35.97

## Supplementary Materials

Supplementary materials can be found at https://www.mdpi.com/1422-0067/20/20/5003/s1.
