# Supplementary material for: Effects of Dietary Bisphenol A on the Reproductive Function of Gilthead Sea Bream (Sparus aurata) Testes
Source: Int J Mol Sci. 2019 Oct 10;20(20):5003. doi: 10.3390/ijms20205003 (PMC6835794; doi:10.3390/ijms20205003)
Supplement: Supplementary file 1 [file ijms-20-05003-s001.pdf]

International Journal of *Molecular Sciences*

MDPI

Article

Effects of Dietary Bisphenol A on the Reproductive Function of Gilthead Sea Bream (*Sparus aurata*) Testes

Isabel Forner-Piquer[1], Ioannis Fakriadis[2], Constantinos C Mylonas[2], Fabiana Piscitelli[3], Vincenzo Di Marzo[3,4], Francesca Maradonna[1], Josep Calduch-Giner[5], Jaume Pérez-Sánchez[5], Oliana Carnevali[1*]

Table S1. Supplementary material. Primer list for real-time PCR.

Gene name	Symbol	GenBank Accession number	Tissue	Primer sequence
**Endocannabinoid receptor type I**	*cnr1*	MG570167	Testis	GCT CAA CTC CAC CGT CAA CCC AAT C
				CCG CAG GTC CTT GCT CCT CAAC
**Endocannabinoid receptor type II**	*cnr2*	MG570168	Testis	TGG TCC TCA CGA AGG CGG AGA AGA
				CCA GCC AGC AAA CAG ATA GAG CCA ATG AC
**Peroxisome Proliferator activated receptor α**	*pparα*	AY590299	Testis	TCT CTT CAG CCC ACC ATC CC
				ATC CCA GCG TGT CGT CTC C
**Peroxisome Proliferator activated receptor β**	*pparβ*	AY590301	Testis	AGG CGA GGG AGG AGT GAG GGA TGA GGG GAG
				CTG TTC TGA AAG CGA GGG TGA CGA TGT TTG
**Peroxisome Proliferator activated receptor γ**	*pparγ*	AY590304	Testis	CGC CGT GGA CCT GTC AGA GC
				GGA ATG GAT GGA GGA GGA GGA GAT GG
**Transient receptor potential cation channel subfamily V member I**	*trpv1*	MG570174	Testis	GGG TCT ACC TCC TGT ACC TAG TCA TCT TCA C
				GCC AAA CGG ATT CTT TC CAT CCC TCC TAT T
**Fatty acid amide hydrolase**	*faah*	MG570169	Testis	CCT CTG GCT CCT CGT ATT GCT TCC
				CAA CCG ATC CCA CTC CAC AGA TAG C
**N-acyl phosphatidylethanolamine phospholipase D**	*nape-pld*	MG570170	Testis	CGG CTA CTG CTC TTC CTT CCA
				TGC GAG GTC AAC GGG TCC ATA
**Abhydrolase Domain Containing 4**	*abdh4*	MG570171	Testis	GTG ACC CTA CCA AAC CAG GAT CGA ATA TG
				CGC TTC GCC TTG TTG GTG ACG
**Cytosolic phospholipase A2**	*cyt-pla2*	JX975709	Testis	GCT GAT GAA CAG AGT GAG CAG TAA CC
				GCC TGG ATG TAG TTG GTG ATG TGT
**Cyclooxigenase 2**	*cox2*	MG570172	Testis	GCG GAC GGG ATA TAC TGG ACA A
				GGC GAA GGT TTC AGG GAG ATT T
**Diacylglycerol lipase alpha**	*daglα*	MG570173	Testis	GCG TCT GGT TGG TGG TTC TG
				AGG ACT GGT CTG AGC CAT ACG
**Monoacylglycerol lipase abdh6a**	*abdh 6a*	JX975713	Testis	CAT CAC CTC CGT CCT GTT C
				CGC CAG TAC CAG TTG TAG G
**Monoacylglycerol lipase abhd12b**	*abdh 12b*	JX975712	Testis	CGT GCT GGT GCC TGA CTA C
				CGG CTT CAG TCG GCT CAC
**Leptin receptor**	*lepr*	MG570178	Testis	GGC GGA ACT GAT TCT ACT CTG
				AGT ATC GGA CCT CGT ATC TCA

**Leptin a**	*lepa*	MG570179	Testis	CAG CCT GAT CTC AGA CGA CCT TGA CAA C<br>TGA TCC AGG AAT CCA GAC AGC GAA GA
**Estrogen receptor alpha**	*era*	AF136979	Testis	ACT CAT CTT CGC ACA GGA CCT CAT ACT<br>GCC TTC AAC ACA GTC GCC CTC A
**Estrogen receptor beta**	*erb*	AF136980	Testis	GAG GCG AGT GAC CCG TCT GT<br>CAC GCT TGG TCC GCT AGT TCT G
**Progesterone receptor**	*pr*	JQ692980	Testis	CAG CTT CCG AAG GTC CTG GC<br>GTG TGG AAC AAA AGC GGC CTC A
**Androgen receptor**	*ar*	JN412131	Testis	GCT GAG GTC GCC ATA TCC CAA CT<br>GCC ACT CGC TGA CTT CAC TCT TCA
**Luteinizing hormone receptor**	*lhr*	MG700544	Testis	GCA GGA CCG AGA TCC GTG ACA<br>GGG ACC TCT TTC AGT GGC AGA TGA
**Follicle – stimulating hormone receptor**	*fshr*	Y587262	Testis	CAC CCC GAC CCA AGA CGA AT<br>TAG TGG GAC GGC GGA CAT GA
**Gonadotropin releasing hormone receptor**	*gnrhr*	MG700545	Testis	CGT CTG ACC TCG ACA ACA ACA CCG TTA T<br>CAA AGA GTT GGC GGC ACA CG
**17b hydroxysteroid dehydrogenase 14**	*17b-hsd*	MG700546	Testis	CTG TCA CGG TGG AGC ATT AC<br>GTG GAT TTA TGA GGC GGG TG
**3b hydroxysteroid dehydrogenase delta**	*3b-hsd*	MG700547	Testis	ACC GTC TTC CAC ATC GCA TCC AT<br>TTG AAT ACA CGC CTC CAG AAG CAG C
**Vitellogenin a**	*vtga*	HG794235	Liver	CTA AGG GGA GCC ACA GCA TAC AAC TAC A<br>CTG CCT CAA GGA TCA CAA TAC CAC TAG C
**Zona pellucida like domain containing protein 1**	*zp1*	MG700543	Liver	AAT TAC ACC AAG CCC CTG AC<br>CGA CCA CCT GAA CGT AGA TG
**Zona pellucida sperm-binding protein 3**	*zp3*	X93306	Liver	TAG GCT CAC AGG CTC AG ACT CTA AGT TCA T<br>CCA ACT GGA ACC GCA GCA CGT TAT CT
**β-actin**	*actb*	X89920	Liver, testis	TCC TGC GGA ATC CAT GAG A<br>GAC GTC GCA CTT CAT GAT GCT
